# Epilepsy, and Other Chronic Convulsive Diseases

**Published:** 1885-12

**Authors:** 


EPILEPSY AND OTHER CHRONIC CONVULSIVE DISEASES. Their Causes, Symptoms and Treatment. By W. R. Gowers, M. D., F. R. C. P., Assistant Professor of Clinical Medicine in University College, Senior Assistant Physician to University College Hospital, Physician to the National Hospital for the Paralyzed and Epileptic. New York: William Wood & Co. 1885.

This work is a contribution to the progressive study of nervous diseases, and the author states that in any case of convulsions the first step in diagnosis must be to ascertain whether the convulsion is epileptic or hysteroid, and if there is hysteroid convulsion, whether this occurs alone or succeeds an epileptic fit. If other explanation does not appear, the fits are probably due to idiopathic epilepsy, and the probability of this is increased if any history can be obtained of inherited tendency, or that convulsions or attacks of petit mal have occurred in earlier life. To effect a cure of this disease, it is most important to maintain, by long treatment, the initial arrest of fits, which may be affected by various agencies, amongst which the bromide of potash holds a prominent place. The shower-bath, with other tonic measures, are also recommended, without recognizing the advantages claimed by Dr. Pepper for confinement to bed and systematic feeding during the treatment of this disease.

Barring the sixty-four pages of advertisements at the close of the volume, this September issue is in keeping with the usual good standard of the publishers.

